# Rare heterotopic pregnancy after frozen embryo transfer: a case report and literature review

**DOI:** 10.1186/s12884-020-03214-1

**Published:** 2020-09-17

**Authors:** Xianping Wang, Ding Ma, Yangang Zhang, Yanhua Chen, Yuxia Zhang, Zhongyu Liu, Xingyu Bi, Xueqing Wu, Junmei Fan

**Affiliations:** 1 Children’s Hospital of Shanxi and Women Health Center of Shanxi, Department of Reproductive Medicine Center, Taiyuan, Shanxi China; 2 Shanxi Bethune Hospital, Department of Urology, Taiyuan, Shanxi China; 3 Department of Obstetrics and Gynecology, Third Hospital, Peking University, Beijing, China

**Keywords:** Heterotopic pregnancy, Retroperitoneal, Laparotomy, Case report, Frozen embryo transfer

## Abstract

**Background:**

Heterotopic pregnancy occurred after frozen embryo transfer with two D3 embryos, and the case had a history of bilateral salpingectomy due to salpingocyesis. An ectopic heterotopic pregnancy was implanted in the left psoas major muscle, which has not been previously reported.

**Case presentation:**

A 33-year-old woman presented with left back pain after curettage due to foetal arrest in the uterus without vaginal bleeding and spotting, and painkillers relieved the pain initially. When the painkillers ceased to work, the patient returned to the hospital. The β-human chorionic gonadotropin (β-hCG) level remained increased compared with the time of curettage, and a diagnosis of retroperitoneal abdominal pregnancy was suggested by ultrasonography and computerized tomography (CT) with the gestational sac implanted in the left psoas major muscle at the left hilum level. Laparotomy was performed to remove the ectopic pregnancy. During the operation, we carefully separated the adipose tissue between the space of the left kidney door and left psoas major muscle, peeled away the gestational sac that was approximately 50 mm × 40 mm with a 25-mm-long foetal bud, and gave a local injection of 10 mg of methotrexate in the psoas major muscle. Fifty days later, β-hCG decreased to normal levels.

**Conclusion:**

It is necessary to pay more attention to the main complaints to exclude rare types of ectopic pregnancies of the pelvis and abdomen after embryo transfer.

## Background

Ectopic pregnancy is one of the most common acute abdominal diseases in obstetrics and gynaecology. It refers to a pregnancy in which the gestational sac is implanted outside of the uterine cavity, and the incidence of ectopic pregnancy is 2 ~ 3% [1]. Abdominal pregnancy accounts for 1% of ectopic pregnancies, and retroperitoneal pregnancy is a rare type of abdominal pregnancy, so only a few cases have been reported worldwide [2]. However, the mortality of intraperitoneal pregnancy was 8 times higher than that of common pregnancy [3]. Heterotopic pregnancy refers to the coexistence of intrauterine pregnancy and ectopic pregnancy. With the development of IVF-assisted pregnancy technology, the incidence of heterotopic pregnancy has been increasing, accounting for 1% of IVF pregnancies [4]. This report describes a case of heterotopic pregnancy that occurred after embryo transfer, with a history of bilateral salpingectomy due to salpingocyesis. The ectopic pregnancy was implanted in the left psoas major muscle, which was not previously reported [5]. Patient written consent was obtained, and the protocol was approved by the Ethics Committee of Shanxi Maternal and Child Health Care Hospital (IRB-KY-2017).

## Case presentation

**Fig. 1:**
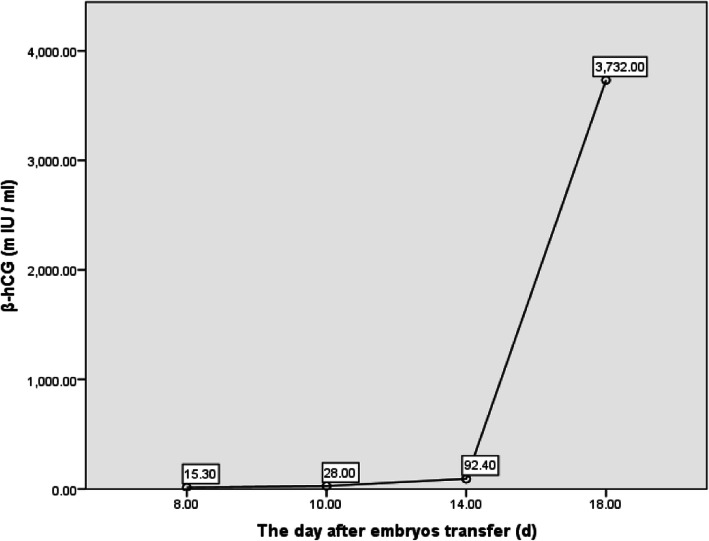
The β-hCG changes after embryo transfer

**Fig. 2:**
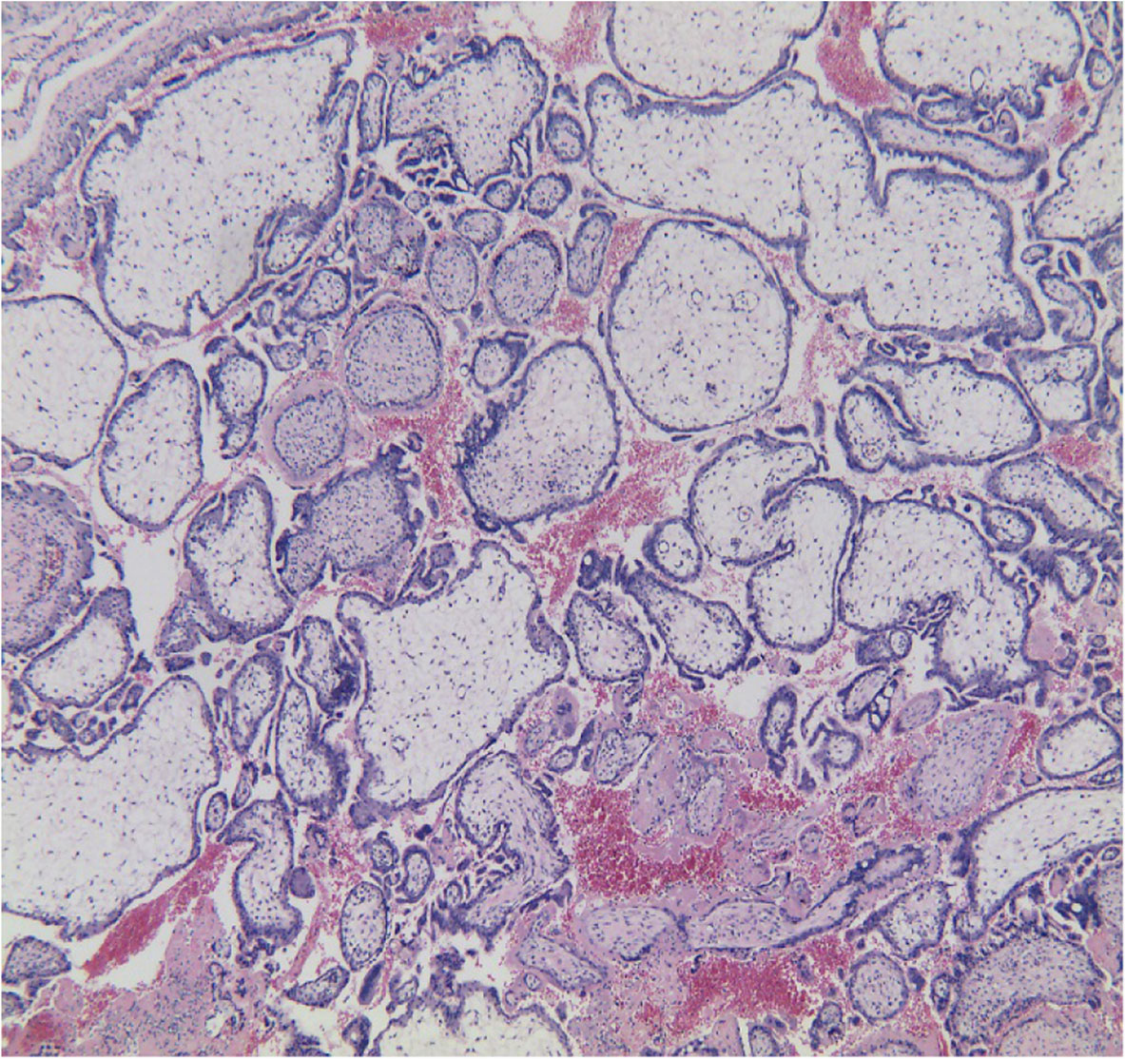
Pathological result of curettage tissue (40 X)

**Fig. 3:**
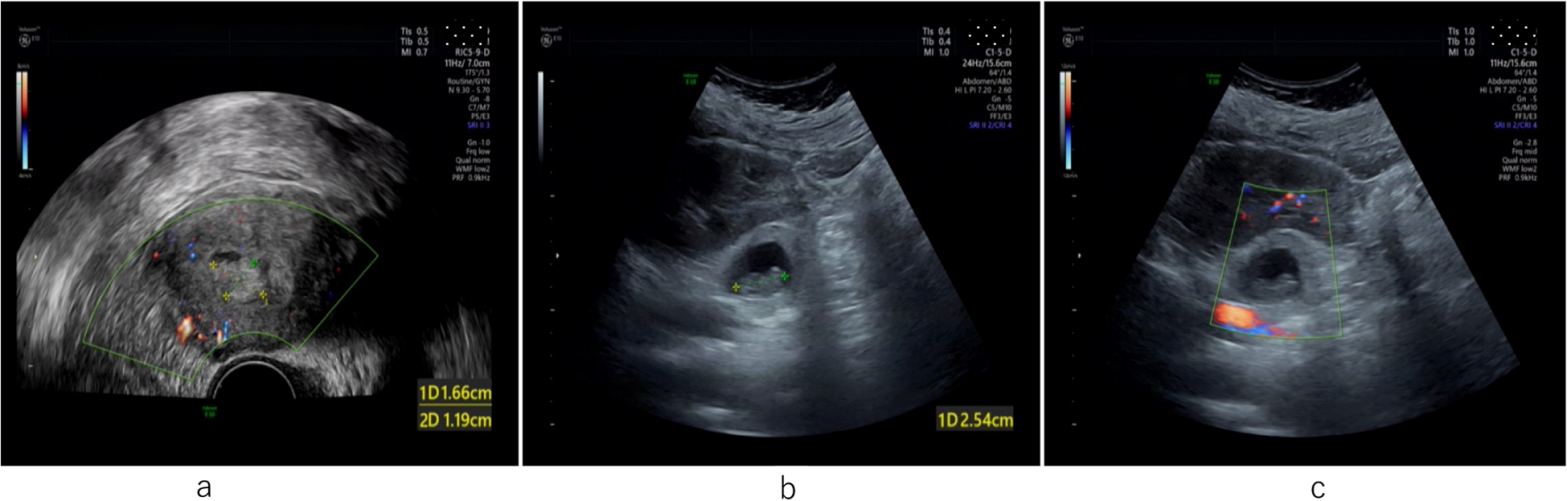
The ultrasonograms before the laparotomy . **a** transvaginal ultrasonogram of uterus after curettage; **b**: the feotal bud of retroperitoneal pregnancy by transabdominal ultrasonogram; **c**: transabdominal colour Doppler ultrasonogram of retroperitoneal pregnancy

**Fig. 4:**
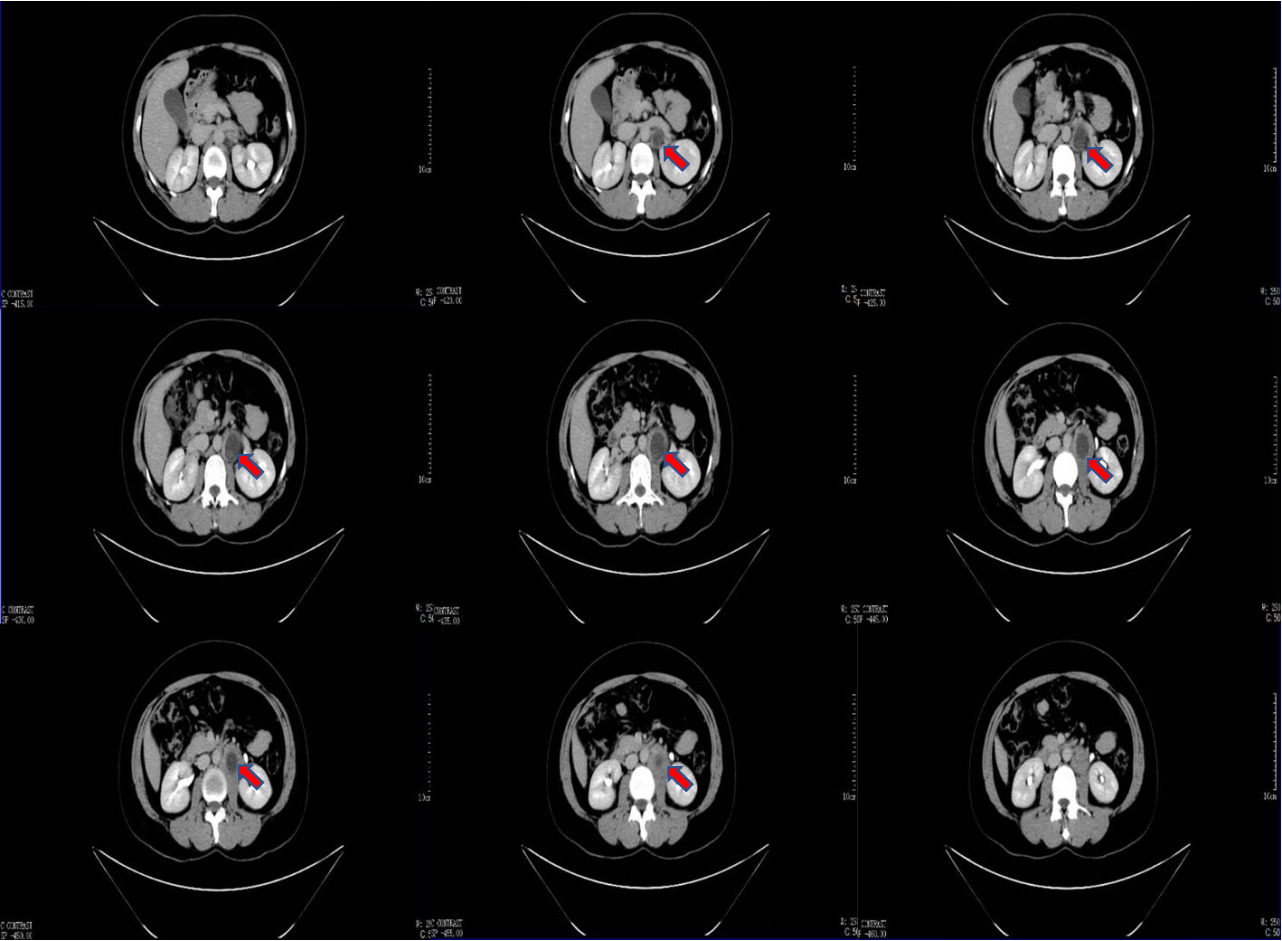
Abdominal computed tomography of Axial plane. The cystic mass was indicated by red arrow was gestational sac

A 33-year-old pregnant woman, gravida 6, para 1, was hospitalized on August 10, 2019, 52 days after embryo transfer, presenting with 11 days of pain in the left lumbar back after curettage. The menstrual cycle was 29–30 days, 3–5 days, LMP: 2019-6-8. On June 19, two D_3_ embryos (10C, 2; 8C, 2) were thawed and transferred in the natural cycle. After transfer, luteal support was provided, including diprogesterone (Abbott, the Netherlands) 20 mg, b.i.d., p.o. and progesterone (Xianju, China) 40 mg, q.d., i.m. The serum β-hCG increased from 8 days after embryo transfer (Fig. 1). Twenty days after transfer, the anechoic area in the uterus was 20 mm × 10 mm by vaginal pelvic ultrasound, similar to the gestational sac. Thirty-five days after transfer, the anechoic area in the uterus was re-examined and found to be 19 mm × 6 mm, and accessory tissues showed no obvious abnormalities. Both laboratory data and ultrasound imaging results suggested embryo arrest, and curettage was performed on July 30 (41 days after transfer). Villus tissue was observed in the uterine cavity after the operation. On August 2nd, the patient experienced intermittent left back pain with distension and no relief. The patient took painkillers, and the pain was relieved without dizziness, anal distention, nausea or vomiting. On August 9th, the pathological result of curettage content validated the villous tissue supporting uterus pregnancy (Fig. 2). On August 10th, the patient experienced worse pain that was not relieved with painkillers. The vital signs of the patient were steady, and percussion pain of the left costal ridge angle was positive. The ultrasound of the pelvic cavity showed that the uterus was 49 mm × 55 mm × 44 mm with regular shape, uniform echo of the myometrium, and a 17 mm × 12 mm uneven echo area in the uterine cavity (Fig. 3 a). Colour Doppler flow imaging showed a small amount of blood flow, 25 mm × 12 mm in the left ovary and 27 mm × 15 mm in the right ovary. No echoic area was found in the Douglas pouch. Behind the left kidney, there was a 49 mm × 39 mm heterogeneous echo area, which was similar to the gestational sac, a 36 mm × 25 mm anechoic area. The crown-lump length of the embryo was 25 mm, without primitive beating of heart tube, suggesting abdominal pregnancy (Fig. 3 b/c). The urgent β-hCG blood test was 74,678 mIU/ml. Retroperitoneal pregnancy was hinted. Then, abdominal CT showed that a round soft tissue density shadow could be seen in front of the left psoas major muscle, with dense liquid in the centre. The maximum diameter of the lesion was 36 mm, and the maximum diameter of the low density was 25.7 mm. The arteriovenous phase could be seen with venules entering. Occupying lesions of the left psoas major muscle at the middle and lower poles of the left kidney were considered (Fig. 4), and the diagnosis of ectopic pregnancy was established in combination with the history.

The patient had a spontaneous abortion in 2009, and a right salpingectomy was performed in 2010 due to an ectopic pregnancy of the right fallopian tube. In 2011, a baby girl was delivered vaginally at full term. In July 2012, an ectopic pregnancy of the left fallopian was treated with drugs. In 2013, salpingography showed that the left fallopian tube was unobstructed and that the right was absent. In June 2013, in vitro fertilization was performed, two D_3_ embryos were transferred, and laparoscopic left salpingectomy was performed due to the left fallopian pregnancy. In April 2019, two blastocysts were transferred in the natural cycle without pregnancy.

**Fig. 5:**
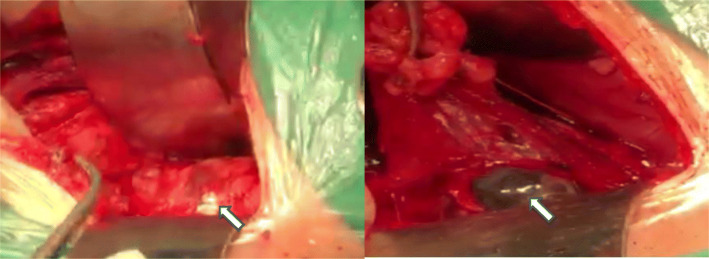
The laparotomy view, the cyst mass implanted in left psoas major muscle (arrow)

**Fig. 6:**
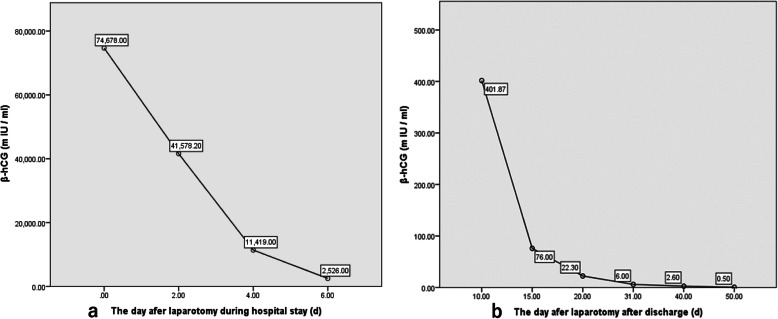
The β-hCG changes after laparotomy

After admission, laparotomy was performed with a 15-cm-long incision between the 11th costal segment of the posterior axillary line, which was then gradually separated into the lower back fascia layer by layer, freed and removed to the 11th ribs to fully expose the operative field. Careful blunt separation of the retroperitoneal space was performed. At the position of the left renal hilum, a gestational sac implanted in the left psoas major muscle was exposed, and the capsule was complete. After freeing of the tissue around the gestational sac, the gestational sac with a size of approximately 50 mm × 40 mm was clearly exposed, and the foetal bud was approximately 25 mm long (Fig. 5). Sufficient haemostasis was achieved at the attachment of the gestational sac, and 10 mg of methotrexate was injected locally. Before discharge, the patient expressed remorse for not communicating with doctors regarding the pain at the onset until painkillers were useless and appreciated the diagnosis and treatment in time. Fifty days later, blood serum β-hCG decreased to normal levels (Fig. 6).

## Discussion and conclusions

Heterotopic pregnancy refers to pregnancy coexisting in the uterus and multiple parts outside of the uterus at the same time. The incidence in natural pregnancy is 1/30,000 [6], ovulation induction is 1/900, and assisted reproductive technology is 1/100 [7], while maternal mortality is 5/1,000,000 [8]. Abdominal pregnancy is a rare ectopic pregnancy, accounting for 1% of ectopic pregnancies [9], and the incidence is 1/10,000 ~ 1/3000 [10, 11]. Abdominal pregnancy was reported in the Douglas pouch of the pelvis, serous layer of the uterus, appendages, abdominal organs, liver, omentum, intestine, appendix, retroperitoneal space, abdominal wall, etc. [5]. Rare ectopic pregnancy is usually misdiagnosed in the early stage until bleeding, pain and other symptoms occur [12]. Whether the early intrauterine pregnancy embryo survives or not, the situation of ectopic pregnancy or heterotopic pregnancy might be ignored, and if not diagnosed, these conditions can endanger the patient’s life [13]. The blood β-hCG of the case was positive 8 days after two embryos were transferred and continued to increase. A similar gestational area was observed in the uterine cavity 35 days after transfer, but no embryo bud or yolk sac was found. In consideration of foetal arrest, uterine curettage was performed. Postoperatively, lumbago and back pain symptoms appearing outside the hospital might be caused by ectopic gestational sac growth resulting in stimulation of the nerve of the left psoas major muscle. At the beginning, the patient took painkillers, which led to a missed early diagnosis. According to the clinical symptoms, combined with the auxiliary examination, heterotopic pregnancy was made with the uterus, and retroperitoneal pregnancy was gradually arrested. For heterotopic pregnancy, if the intrauterine embryo survived and was diagnosed early, most of the cases could be successfully preserved by surgery [11]. If the diagnosis of abdominal pregnancy is later than 24 weeks, the mother is healthy, and the foetus is alive, on the basis of the informed consent of the couple, they could be admitted to the hospital to continue pregnancy [14]. However, some scholars held different opinions that abdominal pregnancy once diagnosed should be actively given surgical treatment due to the high mortality rate of mothers and foetuses and the high birth defect rate of new-borns [15].

This case of heterotopic pregnancy occurred after salpingectomy. There are some questions still to be clarified: how did the embryo enter the abdominal cavity from the uterine cavity, even arriving at the space between the retroperitoneal retrorenal fascia and psoas major muscle? How could the embryo implant in the psoas major? It has been reported that iatrogenic intraperitoneal pregnancy and embryo retrograde lead to retroperitoneal pregnancy [16]. No abnormal channels were found in the uterine cavity in hysteroscopy before this pregnancy. The embryo transfer was carried out under the guidance of ultrasound in our reproductive centre, and the CCD hose was selected. The whole transfer process was smooth, and we were aware that the transfer tube did not penetrate the uterus. Therefore, embryos free from the uterine cavity to the abdominal cavity caused by iatrogenic factors could be excluded. The kidney is one of the extraperitoneal organs, and its position is relatively fixed. The retroperitoneal space is the area between the parietal peritoneum and the fascia transversalis of the abdominal posterior wall. The retroperitoneal space is divided into the anterior pararenal space, the perirenal space and the posterior renal fascia space. In this case, the position of the ectopic pregnancy was located in the posterior renal fascia space, that is, between the posterior renal fascia, the lateral vertebral fascia and the fascia transversalis. There were liposomes in the postrenal space. The embryo was implanted in the psoas major muscle within this space, which has not been reported before. We speculated that the bilateral fallopian tube stumps of the patient after resection were spontaneously reperfused and that one embryo moved in a retrograde manner into the abdominal cavity. At the same time, the patient had a previous history of laparotomy and laparoscopic pelvic surgery, which affected the original anatomical structure of the pelvic cavity and abdominal cavity, resulting in abnormal channels between the abdominal cavity and retroperitoneum. The embryo retrogradely moved into the pelvic cavity through an abnormal channel, and under the promotion of intestinal peristaltic waves and body position changes, it arrived at the left renal hilum nearby, where loose pararenal liposomes might also provide necessary nutrients for embryo implantation in the psoas major muscle. Previous studies reported abdominal pregnancy after bilateral salpingectomy and inferred that there was a fistula leading to the abdominal pregnancy [17]. Others reported that during the implantation process after the disappearance of the zona pellucida, the cells with differentiation potential could fall off and be implanted in the capillaries or lymphatics, and with the circulation of blood or lymph, they would continue to grow in the retroperitoneum, resulting in retroperitoneal pregnancy, which is similar to the metastasis mechanism of gynaecological cancer; therefore, the implantation site of ectopic pregnancy distribution was consistent with large vessels in the female pelvic cavity [1, 12].

The ectopic heterotopic pregnancy was implanted in the psoas major muscle, and the length of the embryo bud was 25 mm, which might be related to some key factors in the embryo implantation surroundings. During the process of the embryo moving from the uterus to the abdominal cavity and retroperitoneum, and further to the retrorenal space, and implanting in the psoas major muscle, a series of environments encountered by the embryo cannot be compared to that in the uterus. In surgery, the foetal bud was 25 mm long, which was equivalent to 9 weeks of pregnancy. This meant that the embryo had obtained nutrients in early development, but the specific mechanism of this obtainment was not clear. In addition, previous studies proposed that a history of pelvic surgery and ectopic pregnancy were risk factors for intraperitoneal pregnancy [18, 19]. The case validated this view.

For the management of pregnancy patients after IVF-ET, attention has mostly been paid to the situation of the pelvic cavity to exclude ectopic pregnancy. The clinical manifestations of retroperitoneal pregnancy vary greatly, especially for rare heterotopic pregnancies. In this study, there was no discomfort in the upper abdomen and lumbar back initially. Therefore, the possibility of retroperitoneal pregnancy was ignored, and the diagnosis of heterotopic pregnancy was missed. This case also suggested that we should closely monitor any changes in β-hCG levels in the follow-up after embryo transfer. Especially for two embryo transfers, we should routinely investigate ectopic pregnancy in the pelvic and abdominal cavities, and for patients who undergo uterine curettage due to foetal arrest, we should closely monitor the decline in β-hCG after operation to further exclude rare ectopic pregnancy and trophoblastic diseases. During the process of eliminating ectopic pregnancy, especially for rare ectopic pregnancy, ultrasound, CT and MRI are helpful in determining the existence of ectopic pregnancy and the anatomy around the gestational sac, which provided guidance in making a reasonable treatment plan. Meanwhile, the complaints of patients are important in clinical diagnosis and treatment.

The choice of therapeutic method (including open surgery, laparoscopic surgery and drugs) was based on the haemodynamics stability and the retention of intrauterine pregnancy. Generally, for patients with unstable haemodynamics and intrauterine pregnancy that needs to be simultaneously retained, surgical treatment is generally the first selection. The choice of surgical approach is also related to the experience of the surgeon, the position of the ectopic pregnancy and the experience with laparoscopic operation. However, for those with stable haemodynamics and intrauterine pregnancy that does not need to be preserved, drug treatment might be a choice, such as methotrexate, potassium chloride and hypertonic saline [20]. Singh Y et al. suggested that the placenta should be preserved locally to avoid bleeding and organ damage caused by stripping, but the disadvantage was that the risk of postoperative infection, secondary bleeding and even trophoblastic disease increased [21]. After removing the gestational sac implanted in the psoas major muscle, considering that trophoblasts might invade into the muscle layer, even if it was cleared as far as possible, the risk of bleeding will be greatly increased. Therefore, after gestational sac stripping, intramuscular injection with 10 mg of methotrexate at the gestational sac implanting site was performed to kill residual trophoblasts. After the operation, regular follow-up was given to evaluate the recovery, and the decrease in β-hCG was slower than that in tubal pregnancy. It took 50 days for β-hCG to decrease to negative. Ansong E et al. [5] compared operation alone and operation combined with methotrexate (i.m. 50 mg/m^2^) for abdominal pregnancy, and the results showed that bleeding in the operation alone group was significantly higher, while the hospitalization time of the operation plus methotrexate (i.m. 50 mg/m^2^) group was significantly shortened. Therefore, if the local tissue is rich in blood supply, methotrexate (i.m. 50 mg/m^2^) could be given, especially for gestational sac implantation in rare sites, which could be helpful in killing trophoblast cells, decreasing β-hCG, and reducing relevant complications. The patient appreciated the treatment we provided for retroperitoneal pregnancy and the regular monitoring for recovery.

In summary, although the patient missed an early diagnosis of the rare heterotopic pregnancy, she was treated in time after showing clinical symptoms and obtained an ideal outcome. For pregnancy after embryo transfer in IVF, we should pay attention to the patient’s main complaints and exclude ectopic pregnancy. Ultrasound examination should be commonly combined. If necessary, we could precisely locate the position of pregnancy sac implantation and the anatomy of the surroundings through CT and/or MRI and design a reasonable treatment plan for patient recovery. For rare site ectopic pregnancies, surgery combined with methotrexate is a good method, and how to combine these approaches deserves to be explored.

## Data Availability

All data analyzed during this study are included in this published article.

## Abbreviations

hCG: Human chorionic gonadotropin
IVF-ET: In vitro fertilization-embryo transfer
CT: Computed tomography
MRI: Magnetic resonance imaging
